# Assessment of children suicide attempts frequency in the peripandemic period

**DOI:** 10.3389/fpsyg.2024.1361819

**Published:** 2024-08-08

**Authors:** Łukasz Wiktor, Maria Damps

**Affiliations:** ^1^Department of Trauma and Orthopaedic Surgery, Upper Silesian Children’s Health Centre, Katowice, Poland; ^2^Department of Trauma and Orthopedic Surgery, ZSM Hospital, Chorzów, Poland; ^3^Department of Anaesthesiology and Intensive Care, Upper Silesian Children’s Health Centre, Katowice, Poland

**Keywords:** pandemic, COVID-19, children, trauma center, suicide, school

## Abstract

**Objectives:**

Our study aimed to evaluate patients after suicide attempts treated at the Department of Trauma Surgery for Children in the peripandemic period, assessment of potential risk factors, and the school’s participation as the unit responsible for the prevention of suicidal behavior.

**Materials and methods:**

Retrospective review of the medical database at equal time intervals of 24 months to identify patients treated before and after the COVID-19 was done. Thorough analysis including injury mechanism, medical procedures, history of previous mental disorders or suicidal behavior was performed. Furthermore, results were compared with the Polish police suicide statistics.

**Results:**

Based on our retrospective review we found 4 patients treated in our department before the pandemic and 10 patients treated after COVID-19 outbreak. The group before SARS-Cov-2 era consisted of three girls and one boy with a mean age of 14.97 (12.7–17.6). The group treated in the pandemic crisis consisted of 8 boys and 2 girls, the mean age was 15.49 (10.8–17.2). In the pre-COVID-19 group, 2 out of 4 patients had received psychiatric treatment before, but none had attempted suicide before. In the COVID-19 group, 6 out of 10 patients had previously received psychiatric treatment, moreover 3 of them attempted suicide before. Based on our analysis, the number of individuals who displayed suicidal attempts has raised. Between 2018 and 2021 the largest number of suicides concerned the 13–18 y.o. group, both for the Silesian Voivodeship (*H* = 9.374; *p* = 0.0092) and for the whole country (*H* = 10.203; *p* = 0.0061).

**Conclusion:**

(1) Results of our study indicate that the pandemic may have caused a wide range of negative mental health consequences for young individuals; (2) Suicide attempts in children are often related with high energy trauma; (3) Teachers and school psychologists, as well as medical health providers, should be aware of rising suicide rates among adolescents.

## 1 Introduction

Suicide is one of the top 20 leading causes of death worldwide, accounting for approximately 800,000 deaths annually, which is a serious public health problem (World Health Organization, 2021). Suicide is the fourth leading cause of death for adolescents between 15 and 19 years (World Health Organisation, 2021). Since stress factors play an essential role in the emergence of suicide attempts (SA) and suicidal ideation (SI), they may have been exacerbated by the pandemic, which could have led to an increased number of suicide attempts (Groh et al., 2024). SA is classified as “intentional self-harm” according to the ICD 10 classification. The category includes intentionally committed poisonings or injuries against oneself. An essential criterion for the SA is the intention to kill oneself by initiating or self-harming the body. A nonfatal SA remains the most potent known clinical predictor of eventual suicide (Harris and Barraclough, 1997). SI refers to thinking about or formulating plans for suicide. The ideation exists on a spectrum of intensity, beginning with a general desire to die that lacks any concrete method, plan, intention, or action and progressing to active suicidal ideation, which involves a detailed plan and a determined intent to act on the ideas (Harmer et al., 2024). Young people with SI often do not access mental health services or even seek professional help (Michelmore and Hindley, 2012; Geulayov et al., 2018). There are number of factors that explain this: concerns around confidentiality, fears of stigma from staff and peers, lack of knowledge of who to seek help from (Williams et al., 2022). However, there are a lot of barriers for young people when they try to access mental health support. Recently young people preferred getting support from online community-based services (forums, webchat, or support groups) when have suicidal behavior (Michelmore and Hindley, 2012; Williams et al., 2018). Possibility to stay anonymous is the most important factor that induce young people to seek help on the Internet (Navarro et al., 2019). Furthermore, face-to-face access to psychological and psychiatric services has been hampered by the pandemic outbreak. Due to the COVID-19, cases of suicide have been reported in many countries affected by the pandemic. Infected patients, healthcare providers including those directly engaged to the treatment of patients with SARS-Cov-2; employees of industries affected by the economic crisis, the whole families of deceased are at risk of committing suicide. Apart from psychosocial stressors, individuals with psychiatric disorders and SI before pandemic started are in the high risk of SA. The COVID-19 pandemic started spreading in November 2019 in Wuhan China. The first case of SARS-Cov-2 infection in Poland was found on March 4, 2020. The fear of being infected as well as restrictions applied to limit infection’s spreading have had a negative impact on family life and human relationships. All of these factors caused anxiety and distress to the children and their families. This study aimed to examine prospective differences in admission rates to the Department of Orthopedic Surgery Children’s Trauma Center after SA in the interval of 4 years covering the peripandemic period.

## 2 Materials and methods

We have used the STROBE protocol, designed for retrospective observational studies, to minimize bias and enhance the quality and transparency of the study (von Elm et al., 2007).

To compare the number of patients treated for injuries following suicide attempts, we retrospectively searched the medical database of patients treated at the Department of Orthopedic Surgery Children’s Trauma Center at two-time intervals. Two equal time frames covering the 24-month period before and after the outbreak of the COVID-19 pandemic were analyzed. We assumed March 4, 2020, as the cut-off date, when the first case of infection in Poland was detected. Far-reaching mental health consequences develop with a delay until the stressor’s onset. It is worth emphasizing that postponing the cut-off date by 1 month when the number of people being infected in Poland increased significantly would not affect the obtained results.

The Department of Orthopedic Surgery medical database was searched in two separate time frames. The initial search revealed 1,151 patients hospitalized in the 24 months before March 4, 2020, and 1,269 patients at the same time after that date. In April 2020, only six patients were admitted to the Department due to the sanitary regime and home isolation. For the obtained data, search criteria for external causes of morbidity and mortality have been established following the International Classification of Diseases and Related Health Problems ICD-10: X60-X84 (intentional self-harm). Search queries used were: [X80 – Intentional self-harm by jumping from a high place; X81 – Intentional self-harm by jumping or lying before moving object; X82 – Intentional self-harm by crashing of a motor vehicle; X83 – Intentional self-harm by other specified means; X84 – Intentional self-harm by unspecified means]. Four records were obtained in the pre-COVID-19 and 10 in the post-COVID-19 period. It is worth highlighting that we did not specify the search criteria for the underlying disease diagnosis. We searched for all patients with an intentional self-harm history. Under the above criteria, the data were additionally analyzed in terms of the history of previous suicide attempts: [Y87.0 – Sequelae of intentional self-harm; Z91.5 – Personal history of self-harm]. The documentation of 14 patients has been subjected to a thorough investigation by two authors (Ł.W and M.D). The medical records were studied individually in detail, including ambulance reports from the scene of the accident with a description of the circumstances of the injury, psychological and psychiatric consultations that the patients underwent during their hospital stay, previous psychiatric treatment history, and specialist orthopedic procedures. The mental health part of suicide was initially explored and documented on a rigorous protocol by a psychiatry consultant with experience working with juvenile patients. The study’s authors, in close cooperation with the consulting psychiatrist, carried out a retrospective analysis of medical documentation. Due to the multi-source nature of the verified data, we have minimized any doubts regarding the SA. Furthermore, we compared our results with the police suicide statistics which are available on the website: https://statystyka.policja.pl (https://statystyka.policja.pl/st/wybrane-statystyki/zamachy-samobojcze/63803,Zamachy-samobojcze-od-2017-roku.html).

We analyzed gender, age, trauma mechanism, sustained injuries, medical procedures, length of hospitalization and history of mental disorders.

### 2.1 Statistical analysis

Pearson’s correlation coefficients, Kruskal Wallis test and Dun’s test were used for the statistical analysis. Multivariate data analysis was performed as more than two groups were compared (Topolski and Beza, 2022). For significant Kruskal Wallis analysis, i.e., *p* < 0.05, Dun’s post-hoc test was used. All tests were calculated at a statistical significance level of alpha = 0.05.

The age of children who attempted suicide before and during the pandemic in the study group was compared. For this purpose, the Mann–Whitney *U* statistic was used. Based on data from police statistics, to check whether the number of suicides in individual age groups is different throughout the study period 2017–2021, the Kruskal Wallis test with *post hoc* Duna was used.

All procedures in the study were in accordance with the ethical standards of the institutional and/or national research committee and with the 1964 Helsinki declaration and its later amendments or comparable ethical standards. For the study ethical approval was waived by the Ethics Committee of the Silesian Medical Chamber in Katowice, Poland (ŚIL.KB.1134.2022) due to the retrospective nature of the study.

## 3 Results

**Figure 1:**
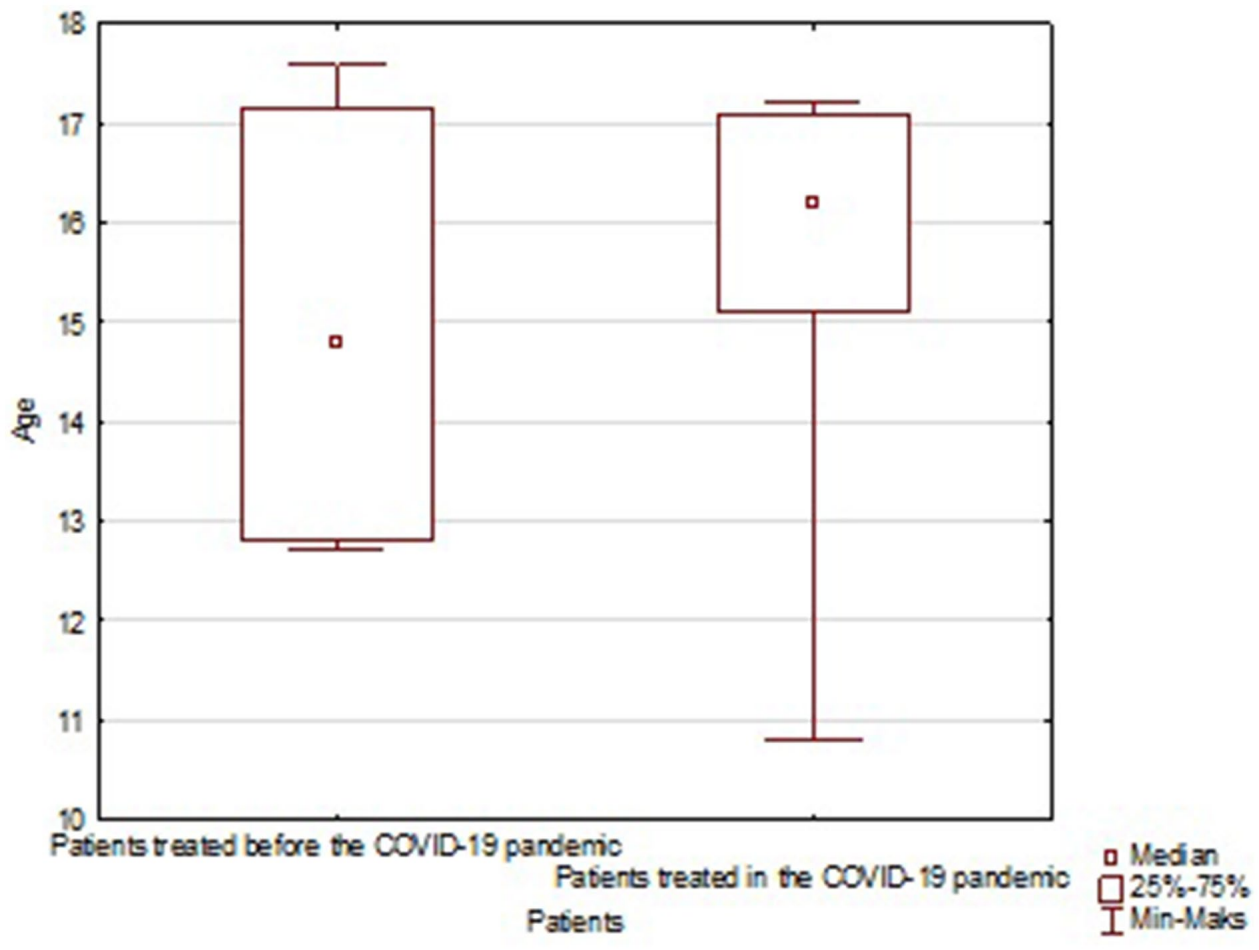
Age distribution of children undertaking SA before and during the pandemic in the study group. Minimal; maximal and average age are marked on the chart.

**Figure 2:**
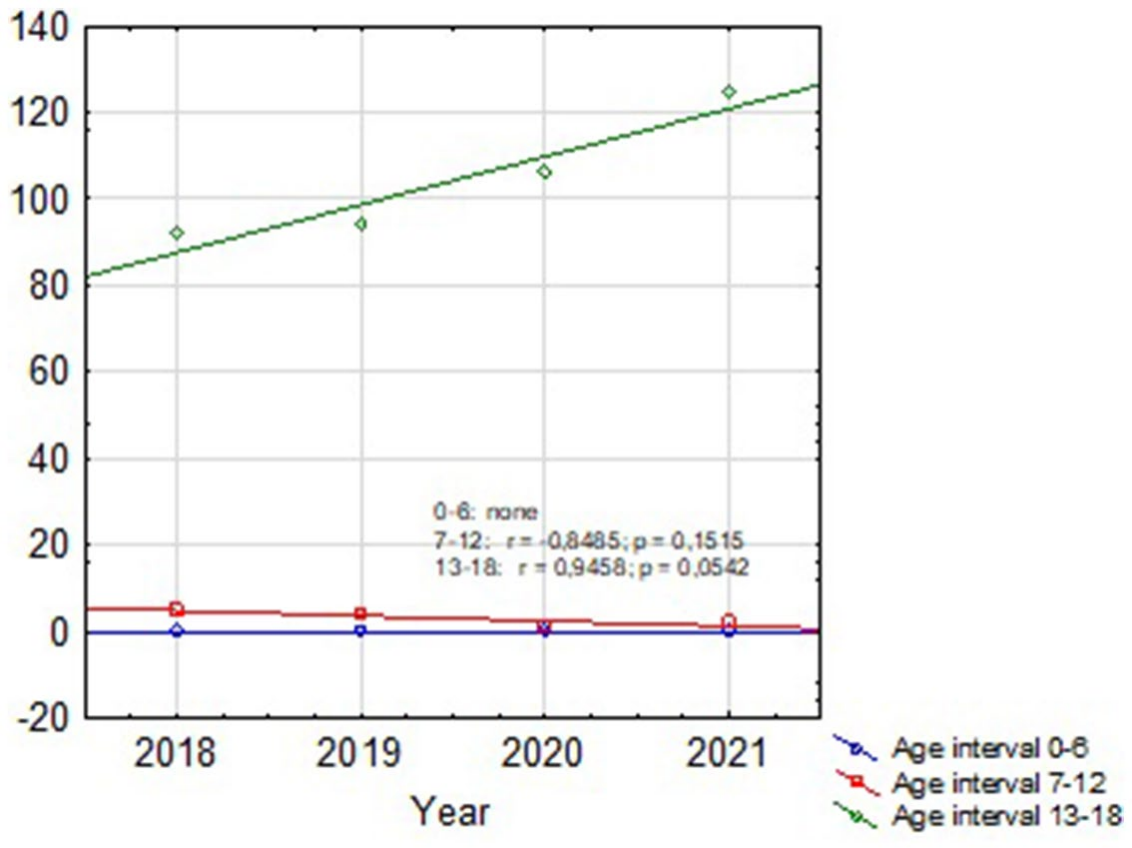
Dispersion chart of the number of suicides by age groups for the Silesian Voivodeship showing increasing trend for SA at 13–18 y.o. group (the number of suicide attempts for each age group in the following years was marked with points).

**Figure 3:**
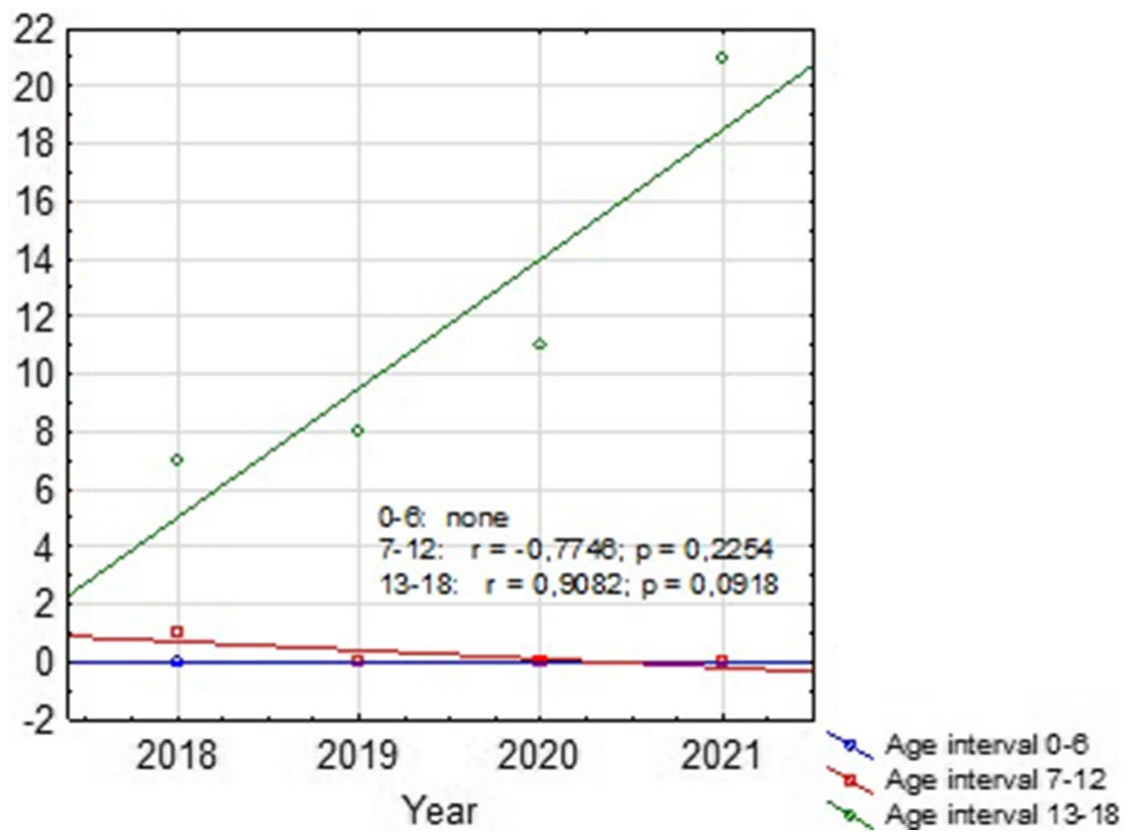
Dispersion chart of the number of suicides by age groups for the whole country showing increasing trend for SA at 13–18 y.o. group (the number of suicide attempts for each age group in the following years was marked with points).

**Figure 4:**
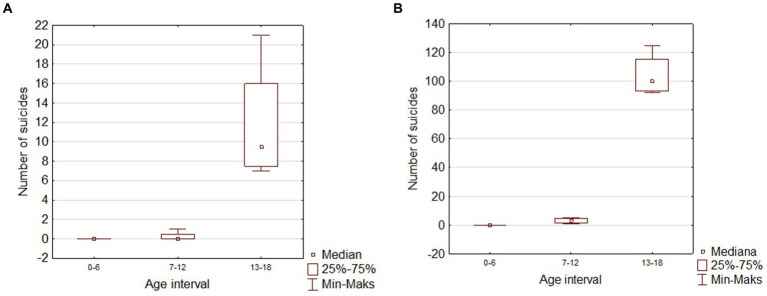
Box and whisker chart of the number of suicides in children in different age groups. **(A)** Silesian Voivodeship; **(B)** Poland.

Based on our retrospective review we found 4 patients treated in our department before the pandemic period and 10 patients treated during COVID-19. The group before SARS-Cov-2 era consisted of three girls and one boy with a mean age of 14.97 (12.7–17.6). The group treated in the pandemic crisis consisted of 8 boys and 2 girls, the mean age was 15.49 (10.8–17.2). In the pre-COVID-19 group, 2 out of 4 patients had received psychiatric treatment before, but none had attempted suicide before. In the COVID-19 group, 6 out of 10 patients had previously received psychiatric treatment, moreover 3 of them attempted suicide before. In 3 patients treated in a pandemic, the suicide attempt was the first manifestation of mental problems, none of these patients received psychiatric help before. The mean hospital stay in both groups was similar and amounted 17.75 days (11–27) for the group before and 16.2 days (3–31) for the group after COVID-19, respectively. The most common SA in the group before COVID-19 was a jump from height. Among the group of patients after COVID-19, we observed 6 jumps from height, one deliberate motor vehicle accident, two throws under the car and one under the train. The study group overview is presented in Tables 1, 2. Descriptive statistics for children with SA before and during the pandemic based on the U Mann Whitney statistic for different groups are shown in Table 3 and Figure 1. According to the police records in the last 4 years no case of fatal SA was found among the 0–6 y.o. group. There was also no increase in SA in the 7–12 y.o. group (only one case in 2018). In 12–18 y.o. interval for the whole country there was an increase of fatal SA (respectively: 2018–92 SA; 2019–94 SA; 2020–106 SA; 2021–125 SA). The same tendency was observed for the Silesian Voivodeship (respectively: 2018–7 SA; 2019–8 SA; 2020–11 SA; 2021–21 SA). Details are presented in Table 4. Figures 2, 3 show trends in the number of suicides by age groups. Although for the 13–18 age group those trends are not statistically significant (*p* > 0.05), we can conclude that at this age the number of suicides increases. Based on data from police statistics, to check whether the number of suicides in individual age groups is different throughout the study period 2017–2021, the Kruskal Wallis test with *post hoc* Duna was used. It can be stated that between 2018 and 2021 the largest number of suicides concerned the 13–18 y.o. group, both for the Silesian Voivodeship (*H* = 9.374; *p* = 0.0092) and for the entire country (*H* = 10.203; *p* = 0.0061). The results are shown on box and whisker chart (Figure 4).

**Table 1:** Patients treated before the COVID-19 pandemic.

No	Sex/age	Sustained injuries	Medical procedures	Hospital stay	Injury mechanism	Previous mental disorders
1	Girl/12.7	Multiple unstable pelvic fracture; middle shaft fractures of the right radius and ulna; psychoorganic syndrome after traumatic encephalitis.	CRIF/anterior external fixation of pelvic ring; CRIF/FIN stabilization of radius and ulna fractures	27 days.	Deliberate jump from high.	Negative history of mental disorders and suicide attempts.
2	Girl/12.9	Fracture of Th12 type A1 AO; left medial and lateral malleoli fractures.	ORIF/screws, K-wires stabilization of malleoli fractures; Jewett brace.	11 days.	Deliberate jump from high.	Psychiatric medications /NA/. Negative history of suicide attempts. Self-harming.
3	Boy/17.6	Fracture of L1 B2, N0, M1 AO type; contusion of L4 and L5; right V metatarsal bone avulsion fracture; abdomen contusions.	Percutaneous transpedicular Th12-L2 fixation.	20 days.	NA	NA
4	Girl/16.7	Bilateral lung contusion; bilateral pneumothorax, fracture of the sacrum, open left humeral shaft with radial nerve laceration fracture of the left humerus.	Left pleural drainage; ORIF / plate fixation of humeral shaft fracture with radial nerve reconstruction (sural cable grafts).	13 days.	Deliberate jump from high.	Mood disturbances. Negative history of suicide attempts.

CRIF, closed reduction with internal fixation; CREF, closed reduction with external fixation; ORIF, open reduction with internal fixation; NA, not available.

**Table 2:** Patients treated in the COVID-19 pandemic.

No	Sex/age	Sustained injuries	Orthopedic procedures	Hospital stay	Injury mechanism	Previous mental disorders
1	Boy/17.1	Unstable fracture of Th8 and Th9 type C; N4; stable C6 fracture type O according to AO; multiple ribs fractures, bilateral pneumothorax, contusion of the right lung; cerebral hematoma, distal phsysis fracture of the left radius.	Transpedicular stabilization of Th6-Th7 / Th10-Th11; laminectomy; right pleural drainage; CRIF of distal phsysis fracture of the left radius – Kwire fixation.	14 days	Deliberate jump from high.	Depression/Pharmacotherapy with sertraline. Appetite disturbances. Self-harming. History of suicide attempts (5x).
2	Boy/16.5	Left femoral shaft fracture; multiple left ribs fractures, left pneumothorax; multiple wounds and abrasions	ORIF/plate fixation of left femoral shaft fracture.	11 days	Deliberate motor vehicle accident.	History of suicide attempts (1x).
3	Boy/15.5	Multifragmentary burst fracture of the right calcaneus; open fracture of left tibia; III, IV, V left metatarsal fractures.	ORIF - open left tibia fracture; CRIF - III, IV, V left metatarsal fractures.	20 days	Deliberate jump from high.	Negative history of mental disorders and suicide attempts.
4	Girl/15.1	Multiple pelvic fractures; proximal physis fracture of right humerus/SH type 2; brain concussion; multiple wounds and abrasions	CRIF/K-wire stabilization of proximal humerus fracture; wounds suturing.	22 days	Throw under the car.	Depression/psychotherapy. Self-harming. Negative history of suicide attempts.
5	Boy/16.1	Stable fracture of Th6; bilateral pneumothorax, bilateral lung contusion; spleen contusion; left humeral shaft fracture; bilateral forearm shafts fractures; walls of the right eye socket fractures	CRIF /FIN stabilization of left humerus; CRIF/K-wires stabilization of right radius fracture; Jewett TLSO.	10 days	Deliberate jump from high.	Negative history of mental disorders and suicide attempts.
6	Girl/16.3	Extensive right trochanteric region wound, damage to the gluteal muscles; open multifragmentary fracture of right tibia and fibula in distal third - type 2 according to GA; multiple pelvic fractures; multiple wounds and abrasions	ORIF/plate fixation of right tibia fracture; suturing the traumatic wound of the right hip; numerous surgical debridement and focal necrosectomy of the right trochanteric area wound.	31 days	Throw under the train.	Mood disturbances. History of alcohol and cigarettes addiction. Self-harming. History of suicide attempts (1x).
7	Boy/17.1	Bilateral open fractures of femoral shaft in distal thirds-type 2 GA; bilateral patella fractures with no displacement; medial condyle fracture of the right tibia with no displacement. Nasal bone fractures; brain concussion	ORIF/external fixation of open femoral fractures; wounds debridement and suturing.	10 days	Throw under the car.	Depression / bipolar disorder. Pharmacotherapy with fluoxetine, sulpiride, lithium. Self-harming (1x). Negative history of suicide attempts.
8	Boy/17.2	Multiple, unstable pelvic fractures; multifragmentary articular fracture of the left distal humerus; multifragmentary fracture of the proximal ulna with a bone lost.	CRIF / anterior external fixation of pelvic ring; ORIF / plate, screws, Weber tension wire of left humeral and ulnar fractures.	28 days	Deliberate jump from high.	Negative history of mental disorders and suicide attempts.
9	Boy/10.8	Bilateral pneumothorax, bilateral lung contusion; open fracture of the distal right radius and ulna-type 1 GA; distal left radius fracture.	ORIF/K-wires stabilization distal right radius and ulna fracture	1-day ICU 2 days	Deliberate jump from high.	Negative history of mental disorders and suicide attempts.
10	Boy/13.2	Severe cardiovascular and respiratory failure; brain contusion and oedema; multiple vertebrae fracture C2, C4, Th9-L5; multiple ribs fractures, bilateral tension pneumothorax, bilateral lung contusion; right femoral neck fracture; bilateral humeral shaft fractures; multiple pelvic fractures. DEATH	CRIF / anterior external fixation of pelvic ring; direct right tibia traction.	13 days ICU	Deliberate jump from high.	Positive psychiatric history (lack of data). Negative history of suicide attempts.

CRIF, closed reduction with internal fixation; CREF, closed reduction with external fixation; ORIF, open reduction with internal fixation; ICU, Intensive Care Unit; NA, not available.

**Table 3:** Descriptive statistics comparing the age of children with suicide attempts before and during the pandemic with a test of differences between the above-mentioned *U* Man Whitney groups.

	Mean	Standard deviation	Median	Confidence −95%	Confidence +95%	Minimum	Maximum
Patients treated before the COVID-19 pandemic	14,98	2,54	14,80	10,93	19,02	12,70	17,60
Patients treated in the COVID-19 pandemic	15,49	2,04	16,20	14,03	16,95	10,80	17,20

*U* Mann–Whitney statistic *Z* = –0.071; *p* = 0.944.

**Table 4:** Police statistics with the number of fatal suicides for whole country and the Silesian province.

Year	Region	Number of fatal suicide attempts	Age interval	Age interval	Age interval
			‘0–6’	‘7–12’	‘13–18’
2017	Silesian Voivodeship	601	0	0	12
2017	Poland	5,276	0	1	115
2018	Silesian Voivodeship	596	0	1	7
2018	Poland	5,182	0	5	92
2019	Silesian Voivodeship	548	0	0	8
2019	Poland	5,255	0	4	94
2020	Silesian Voivodeship	562	0	0	11
2020	Poland	5,165	0	1	106
2021	Silesian Voivodeship	582	0	0	21
2021	Polska	5,201	0	2	125

## 4 Discussion

From the perspective of Children’s Trauma Center exclusively, this paper examines whether the number of suicides in children and adolescents have raised over the past years. The suicide phenomenon is a complex, multifactorial phenomenon that requires interdisciplinary approaches. We evaluated the SA because the number of suicides in children is one of the most decisive factors that measures young people’s mental health condition. Based on our analysis, the frequency of SA among children has raised. Although this increase is not statistically significant (*p* > 0.05), the growing trend is undoubtedly alarming. In the last 4 years at the Children’s Orthopedic Department, we have treated 14 patients after serious SA. The reason for this enlargement is undoubtedly multifactorial, but the pandemic’s contribution could have a substantial impact because the COVID-19 could affect immature populations in multiple fields. Before COVID-19, we have treated 4 patients and after COVID-19, 10 patients which is a more than twofold increase at the same time intervals. 4.45 million people live in the Silesian Voivodeship, which is 12.14% of the country’s population. Our hospital is the only Children’s Trauma Center in the Silesian Voivodeship, which makes us a representative unit. A trauma center with an emergency department cares for immature patients suffering from injuries from the city of Katowice, the capital of the Voivodeship, and the neighboring towns. Through a helipad for receiving patients who have been airlifted to the center from the Silesian region and highly specialized departments with a full range of specialists, the hospital provides professional multidisciplinary care for patients suffering major trauma. At this point, it is also necessary to mention a significant psychiatric problem: self-harm (SH). Self-harming should be divided into nonsuicidal self-injury (NSSI) and suicidal behavior (SB). Prevalence rates of NSSI have been estimated to be around 17% in adolescents (Muehlenkamp et al., 2012). Moreover, assessing the pandemic’s impact on the incidence of NSSI and SB constitutes a field for future analysis. We compared our results with the Polish police suicide statistics additionally. We have isolated the number of fatal SA separately for the entire country and for the Silesian Voivodeship. According to the police records in the last 4 years among children between 12 and 18 y.o. for the whole country there was an increase of SA (92 cases in 2018 compared to 125 cases in 2021). The same tendency was observed for the Silesian Voivodeship (7 cases in 2018 compared to – 21 cases in 2021). In our study only one patient was younger than 12, others were in the 12–18 years interval. Based on the other authors reports, female adolescents have a higher risk of mental disorder, but those studies did not include SI (Ellis et al., 2020; Wang et al., 2020; Zhou et al., 2020). In our group, we observed a predominance of girls taking SA before SARS-Cov-2 era (three girls and one boy) but a definite prevalence of boys in the pandemic crisis (eight boys and only two girls). The pandemic’s physical restrictions and social distancing measures have affected each domain of life. Anxiety, depression, sleep and appetite disturbances, as well as impairment in social interactions are the most common symptoms despite the patient age. The suicide rate in children and adolescents in the last two decades was stable, in contrast to the downward trend in adults (Hepp et al., 2010). Although data from the Swiss National Cohort have shown increasing suicide rate with age: 0 per 100,000 at age 10 years to 14.8 per 100,000 boys and 5.4 per 100,000 girls at 18 years (Steck et al., 2018). Isumi et al. reported that first wave of the COVID-19 has not significantly affected suicide rates among children and adolescents (Isumi et al., 2020). Based on real-time suicide data from multiple countries including Poland, Pirkis et al. relying on an interrupted time-series analysis reported no significant increase in risk of suicide during the pandemics in any country (Pirkis et al., 2021). However, mentioned study did not stratified data by age or gender. Yoshioka E et al. examined the impact of the pandemic on suicide by gender and age through December 2021 in Japan and did not find statistically significant results of the impact of the pandemic on suicide rates for men and women under 20 years old (Yoshioka et al., 2022). Our results could differ from literature reports, probably because our study was limited to patients treated at the Children’s Orthopedic Department exclusively. Interestingly, our observation coincides with the Bruns N et al. reports (Bruns et al., 2022). Based on a retrospective multicenter study among 37 pediatric intensive care units they found that SA increased in adolescent boys (standardized morbidity rate: 1.38) but decreased in adolescent girls (standardized morbidity rate: 0.56).

### 4.1 Initiators of suicide risk

The main initiators which increase the risk for SA and SI in children and adolescents can be divided to school factors – bullying, peer relationships, violent school habitat; family factors – family dysfunction, conflicts, poor communication, and individual psychological factors – depression and other mental health disorders (Picazo-Zappino, 2014; Dilillo et al., 2015; Gunn et al., 2018; Ruiz-Robledillo et al., 2019; Carballo et al., 2020; DeVille et al., 2020). The children reaction for the major stress depends also upon many things including physical and mental health, the socioeconomic family status, cultural environment and experience with previous emergency situations (Smetana et al., 2006; Brooks et al., 2020). What’s interesting, in the study caried with the help of United Nations International Children’s Emergency Fund (UNICEF) based on 1700 children and adolescents from 104 countries they reported that high levels of stress, affect children brain development with long-term consequences (Smetana et al., 2006).

### 4.2 School closure as a stressor related to COVID-19

School closure was a crucial change during the COVID-19 that directly hit young people. Children were unable to meet with friends and teachers, moreover they could not participate in school and out-of-school activities. Staying long-hours at home could affect children in both, positive and negative way. Children may get a reprieve from problems at school: e.g., bullying, negative rivalry, exam stress, peer conflict, romantic breakups, stress related to sexual orientation or on the other hand feel distressed from relationships with family members: i.e., family physical and/or sexual violence, parental alcohol addiction (Klasen et al., 2015; Fegert et al., 2020; Hoekstra, 2020; Chung et al., 2022). G. Segre et al. reported that 78% of the Italian children aged 6 to 14 experienced anxiety symptoms due to the COVID-19 quarantine, and nearly half of the sample (43.9%) reported significant mood symptoms (Segre et al., 2021). Current reports showed a moderate but significant impact of both the sociodemographic and the health risk perception variables related to the COVID-19 experience in the perception of negative and positive feelings (Commodari and La Rosa, 2020; Francisco et al., 2020). It should be pointed out that School closures and isolation from peer groups have also contributed to a significant increase in the number of hours that young people spend online and on social media. There are many proofs that internet overuse can lead to behavioral problems, decrease real-life social interactions, cause relationship disorders and mood dysfunction (Duan et al., 2020; World Economic Forum, 2020). This may correspond with increased risk of suicide, especially in children with pre-existing psychiatric problems. An important aspect is that the school should conduct preventive efforts and spot potential instances of susceptibility. Warning signals that should attract teachers’ attention include increased impulsivity and acts of aggression and violence, rebellious behavior, alcohol or drug abuse, expressing messages about the hopelessness of life and one’s worthlessness, sudden mood changes, and alternating phases of depression and euphoria. If teachers or schoolmates observe the above, the school must immediately act. The lack of psychological and social skills deters teenagers from building good relationships with their environment and meeting essential needs. The intensification of emotions typical of adolescence, including fear and mood swings, increases the risk. The pupil should be referred to a school psychologist or a specialist clinic because a lack of appropriate support can sometimes lead to a state of deep depression. The SI arise as a result of assessing one’s situation as a life trap from which there is no way out. Children with reduced moods tend to take stock of their lives, which is usually negative. Even an insignificant event or a minor setback can become a turning point. Due to the significant variations according to geography, the duration of school closures, isolation, and the availability of resources, the long-term mental health consequences of quarantine in the current context remain uncertain (Almeida et al., 2022). Creswell et al. reported that different groups were affected differently, with preadolescents suffering more than older adolescents, and with those coming from low-income families or having special needs being more affected (Creswell et al., 2021).

### 4.3 Prevention of suicide and suicidal behavior

Short programs focusing on suicide, detached from the real school problems, are ineffective and may even be harmful. Due to the complex and multifactorial determinants of suicide, only an ecological model of prevention can be effective: taking into account both the elimination or weakening of all modifiable risk factors (individual, family, school, community), as well as strengthening protective factors; conducting long-term activities at several levels. School activities should include three levels of prevention (Lazear et al., 2003; Horowitz and Garber, 2006). (I) Health promotion and universal prevention addressed to students, parents, and teachers and including training teachers, creating a supportive environment at school, increasing the self-esteem of children and teenagers, involving students in school projects and events, increasing the educational competences of parents, appointing people to whom they can ask for advice and help. (II) Selective prevention, addressed to groups at increased risk, consisting of collecting information about students’ needs and difficulties, providing support and building motivation, including them in additional programs, developing psychological and social skills, and strengthening cooperation with parents. (III) Indicative prevention, addressed to students from the high-risk group (after a SA or with SI), consisting of care of a school specialist, intervention with the participation of parents, and joint development of a help strategy for the student, referring the student to specialists for an individual diagnosis and possible therapy.

### 4.4 Ethical considerations of conducting retrospective studies on sensitive topics

Understanding and intervening in sensitive areas such as pediatric suicide attempts should depend on detailed analysis. However, research in sensitive areas always requires careful balancing of ethical principles to collect data without harming or jeopardizing individual participants. Research on sensitive topics always raises questions about whether participants experience distress, are sufficiently aware of potential distress to provide informed consent to participate and can accurately predict their level of anticipated distress to make an informed decision to participate. Researchers should be aware of the importance of monitoring participant reactions to the experience of participation using standard measures, such as the Reactions to Research Participation Questionnaire (Newman et al., 2001), and coding responses to open-ended questions. However, retrospective analyses have several limitations owing to their design. In this sensitive field, they can minimize the negative impact of participation in the study on the individual.

## 5 Limitations

There are several limitations to this study. Small study and quite homogenic, narrow population of patients being treated at Children’s Orthopedic Department. We are aware that limiting the study to the trauma center significantly limits its generalizability. However, on the other hand, it allows for the setting to provide unique insights. It addresses the critical and understudied issue of pediatric suicidal behavior among the groups that have not been assessed so far. Another severe limitation related to the small sample size is sampling window, which was skewed towards pandemic. Those mentioned above could affect the interpretation and generalizability of the study findings. The obtained results should be carefully analyzed due to the multifactorial nature of the increased frequency of SA in the pediatric population. Conclusions must be drawn based on the analysis of reports by many authors from different centers. Our study has a retrospective design, which is another limitation. Since the study operations, data collection, and data quality assurance were not planned ahead of time, there are risks of bias, and relying on already collected data could compromise any of these areas. Another limitation is that our current study could not evaluate whether these adverse outcomes will last long after the COVID-19 outbreak. We also did not assess what might be a protective factor that could reduce the risk of SA and SI among the immature population.

## 6 Conclusion

The results of our study, as reported by other authors, indicate that the pandemic may have caused a wide range of negative mental health consequences for young individuals. We found a critical gap in the literature concerning injuries after SA among children and adolescents, however it requires a lot of attention from the whole medical staff. Teachers and school psychologists, as well as medical health providers, should be aware of rising suicide rates among adolescents. Multi-center study should be considered to assess increasing rate of suicide rate among immature population. Furthermore, it is necessary to identify and study other variables that may explain the higher levels of psychological and behavioral symptoms of children and adolescents (e.g., coping strategies and durability of adverse effects). The effort should be put to find effective strategies to promote positive mental health during pandemics crisis in order to reduce suicide rate and suicidal behaviors. Primary and secondary prevention measures are urgently needed to mitigate these effects; otherwise, they can be long-lasting and negatively influence youth development. It is, therefore, imperative to set up real-time and reliable monitoring of suicides and attempted suicides to implement targeted and timely measures and thus accelerate suicide prevention.

## Data Availability

The raw data supporting the conclusions of this article will be made available by the authors, without undue reservation.
